# Percutaneous monoplanar screws versus hybrid fixed axial and polyaxial screws in intermediate screw fixation for traumatic thoracolumbar burst fractures: a case–control study

**DOI:** 10.1186/s13018-024-04547-9

**Published:** 2024-01-22

**Authors:** Yaozheng Han, Jun Ma, Guoquan Zhang, Liangliang Huang, Hui Kang

**Affiliations:** 1 Medical College, Wuhan University of Science and Technology, Wuhan, 430065 Hubei China; 2 Department of Orthopaedic, General Hospital of Central Theater Command, Wuhan, 430070 Hubei China; 3 Medical College, Hubei University of Medicine, Shiyan, 442000 Hubei China

**Keywords:** Thoracolumbar burst fractures, Monoplanar pedicle screws, Hybrid fixed axial and polyaxial pedicle screws, Intermediate screw fixation, Percutaneous pedicle screw fixation

## Abstract

**Background:**

To compare the clinical and radiological outcomes of monoplanar screws (MSs) versus hybrid fixed axial and polyaxial screws (HSs) in percutaneous short-segment intermediate screw fixation (PSISF) for traumatic thoracolumbar burst fractures (TTBFs) in patients without neurologic impairment.

**Methods:**

A consecutive series of 100 patients with single-segment TTBFs and no neurologic impairment who underwent PSISF with 6 monoplanar screws (MS group) or correct were retrospectively enrolled. The demographic data, radiologic evaluation indicators, perioperative indicators and clinical assessment indicators were analysed between the MS group and HS group.

**Results:**

The demographic data and perioperative indicators were not significantly different in the two groups (*P* > 0.05). The postoperative anterior vertebral height ratio (AVHR), kyphosis Cobb angle (KCA), vertebral wedge angle (VWA) and spinal canal encroachment rate (SCER) were significantly improved in both groups (*P < 0.05). The MS group obtained better correction than the HS group in terms of improvement in the AVHR, KCA and VWA after surgery (*P < 0.05). At the last follow-up, the MS group had less correction loss of AVHR, KCA and VWA (*P < 0.05). The MS group presented greater improvement in the SCER at the last follow-up (*P < 0.05). The visual analogue scale (VAS) score and Oswestry Disability Index (ODI) score of all patients were significantly better postoperatively than those preoperatively (*P < 0.05), and the scores collected at each follow-up visit did not differ significantly between the two groups (*P* > 0.05). In the MS group, no internal fixation failure was observed during the follow-up period, but, in the HS group, two cases of internal fixation failure were observed at the last follow-up (one case of rod loosening and one case of screw breakage).

**Conclusions:**

Both MSs and HSs fixation are effective treatments for TTBFs and have comparable clinical outcomes. In contrast, MSs fixation can improve the correction effect, better improve the SCER, and further reduce correction loss as well as reduce the incidence of instrumentation failure. Therefore, MSs fixation might be a better option for treating TTBFs in patients without neurological deficits.

## Introduction

Traumatic spinal fracture accounts for approximately 5–14% of all fractures and is usually caused by high-energy violent injuries such as unintentional falls and traffic accidents [1–5]. A traumatic spinal fracture can compromise the stability of the spine, compress the spinal cord and cause spinal nerve damage [3, 5]. The thoracolumbar junction (T11–L2) is the area at which stress is concentrated, thereby making it prone to fractures, accounting for 71.69% of all traumatic spine fractures [2, 6]. In recent years, percutaneous short-segment intermediate screw fixation (PSISF) has been increasingly performed for the treatment of traumatic thoracolumbar burst fractures (TTBFs) [7–9]. PSISF for the treatment of TTBFs involves inserting screws in the fractured segment and two adjacent segments, forming a 6-screw structure [10]. Previous biomechanical studies have demonstrated that the 6-screw structure clearly increased the stiffness and stability of the internal fixation system during flexion‐extension and lateral bending compared with the traditional 4-screw construct [11, 12]. Similarly, clinical studies have shown that PSISF achieved superior correction and maintenance of the correction compared with percutaneous pedicle screw fixation (PPSF) with 4 screws [13–15].

**Fig. 1:**
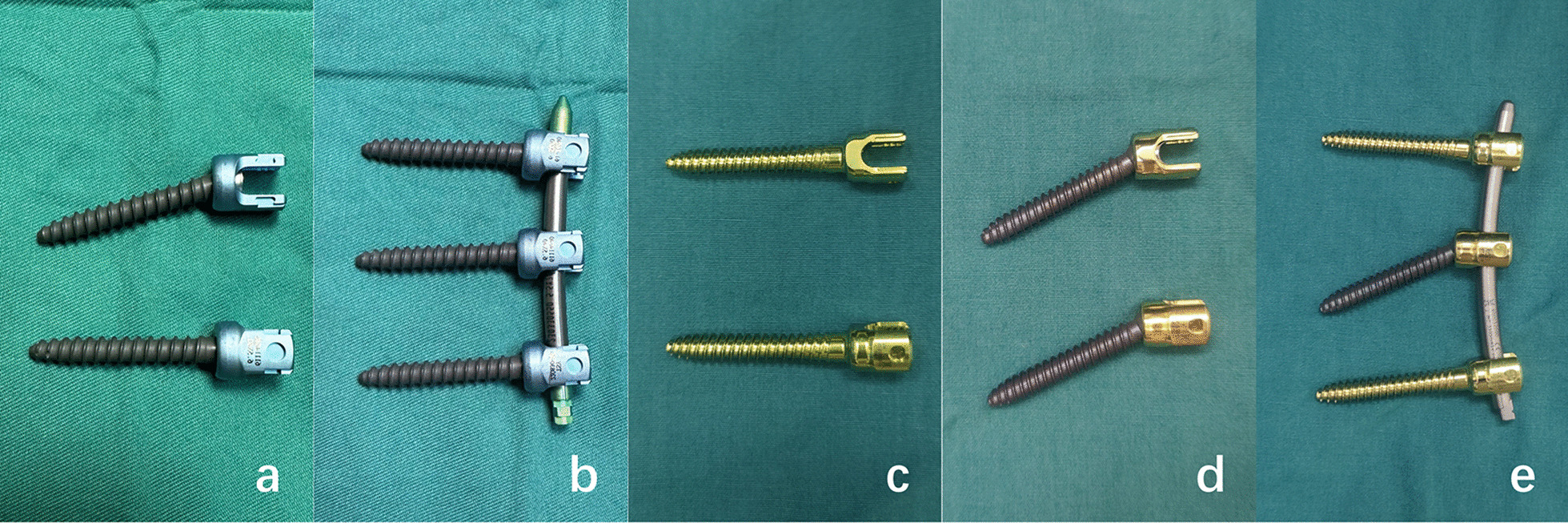
Three pedicle screws and the two new types of fixation methods. **a** MS can swivel freely in the coronal view and can be fixed in the sagittal view. **b** Three robust MSs can provide rigidity in the sagittal view and can smoothly accomplished installation of the connecting rod. **c** Fixed axial screw can be fixed in both the sagittal view and the coronal view. **d** Polyaxial screw can swivel freely in both the coronal view and the sagittal view. **e** Fixed axial screws of the upper and lower can provide firm fixation, and intermediate fixation with polyaxial screw is convenient for rod implantation

Since the PPSF technique was first reported by Magerl, several pedicle screws have been introduced in PPSF including fixed axial screw (Fig. 1c), polyaxial screw (Fig. 1d) and monoplanar screw (MS) (Fig. 1a) [16, 17]. Polyaxial screw is widely utilized in PSISF because of their convenience for rod insertion [18, 19]. Nevertheless, polyaxial screw fixation performed poor results in correcting deformities and maintaining reduction compared to the fixed axial screw and monoplanar screw [18–20]. Fixed axial screw helps improve rigidity due to their structural properties, which is more conducive for restoring the injured vertebral height and correcting kyphosis [21]. However, if the ipsilateral fixed axial screws are not highly aligned in PSISF, percutaneous insertion of the rod becomes difficult [22]. Recently, an innovative MS screw has been developed which combined the advantages of fixedaxial screw and polyaxial screw [18].

To better correct the kyphotic deformity, reduce the loss of reduction, and reserve the convenience of installing the connecting rod, some propose percutaneous MSs fixation of TTBFs [17, 18] (Fig. 1b). Some have suggested that hybrid fixed axial and polyaxial screws (HSs) fixation (Fig. 1e) may be a better option [23]. No relevant studies have reported whether there are differences between both treatments. The purpose of our study is designed to compare the clinical outcomes of MSs versus HSs fixation of TTBFs.

## Methods

### Demographics

From January 2017 to June 2021, a consecutive series of 100 patients with single-segment TTBFs without neurological deficits who underwent PSISF with 6 MSs (MS group) or hybrid 4 fixed axial screws and 2 polyaxial screws (HS group) were retrospectively enrolled. Sex, age, cause of injury, fracture level, AO Spine Injury Classification (AO classification) [24], Thoracolumbar Injury Classification and Severity Score (TLICS score) [25], Load Sharing Classification (LSC score) [26], number of spinal canal encroachment and time of last follow-up were recorded.

Inclusion criteria: (1) between 18 and 60 years of age; (2) with a single-segment vertebral fracture of the thoracolumbar spine involving T11-L2; (3) fresh traumatic fracture; (4) TLICS score greater than or equal to 4; (5) underwent all preoperative, postoperative and follow-up imaging examinations in our hospital; (6) intact pedicles of the injured vertebra; and (7) patients who signed informed consent forms.

Exclusion criteria: (1) time from trauma to surgery more than 14 days; (2) pathological fractures (including tuberculosis, primary or metastatic tumours, etc.); (3) infection; (4) prior spine surgery; (5) congenital spinal deformities; (6) osteoporosis; and (7) incomplete clinical data.

The study was reviewed and approved by the Ethics Committee of General Hospital of Central Theater Command ([2022]060-01) and was performed in conformity with the Declaration of Helsinki. Observational studies were reported using STROBE guidelines [27].

### Surgical procedures

All operations were performed by the same team, and the chief surgeon was an experienced deputy chief physician in the same department. The patients were placed in the prone position on the Jackson surgical table, and the abdomen was suspended, the operating table was bent into a V shape, manual reduction was performed by applying firm pressure on the spinous process adjacent to the injured vertebra. After marking the puncture points approximately 1.5 cm lateral to the markers positioned at the pedicles of the injured vertebra and two adjacent vertebrae, 6 Jamshidi needles were inserted into the corresponding pedicle with proper orientation and to the appropriate depth. After successful puncture, the needle core was withdrawn. After the long guide wire was placed into the anterior medial third of the vertebral body, the screw was implanted. In the MS group, 6 MSs (Shanghai SANYOU, China) were implanted parallel to the upper endplate of the injured vertebra and two adjacent vertebrae. In the HS group, 4 fixed axial screws (Shandong WEGAO, China) were implanted parallel to the upper endplate of the adjacent vertebrae of the injured vertebra, and 2 intermediate short polyaxial screws (Shandong WEGAO, China) were implanted parallel to the pedicle of the injured vertebrae. The connecting rod, with an appropriate length and radians, was inserted after insertion of all 6 screws. In the MS group, during the distraction process, the intermediate MSs were exerted an upward force on the upper endplate of the injured vertebra, aiming to better restore the anterior and middle columns. In the HS group, indirect reduction of the restoration of the anterior column of the injured vertebra by applying appropriate compression to the fixed axial screw-rod system at both ends. All pedicle screws were placed via a minimally invasive percutaneous and inserted into the corresponding segment vertebral body. All above operations were conducted under the guidance of C-arm fluoroscopy.

### Postoperative management and follow-up schedule

For all patients, functional exercises for both lower limbs were started 1 day after surgery. They were encouraged to regularly ambulate 3 days after surgery. All participants were followed up postoperatively for at least 12 months.

### Radiologic evaluation and clinical assessment

Radiologic evaluation indicators, perioperative indicators, and clinical assessment indicators were recorded and assessed.

**Fig. 2:**
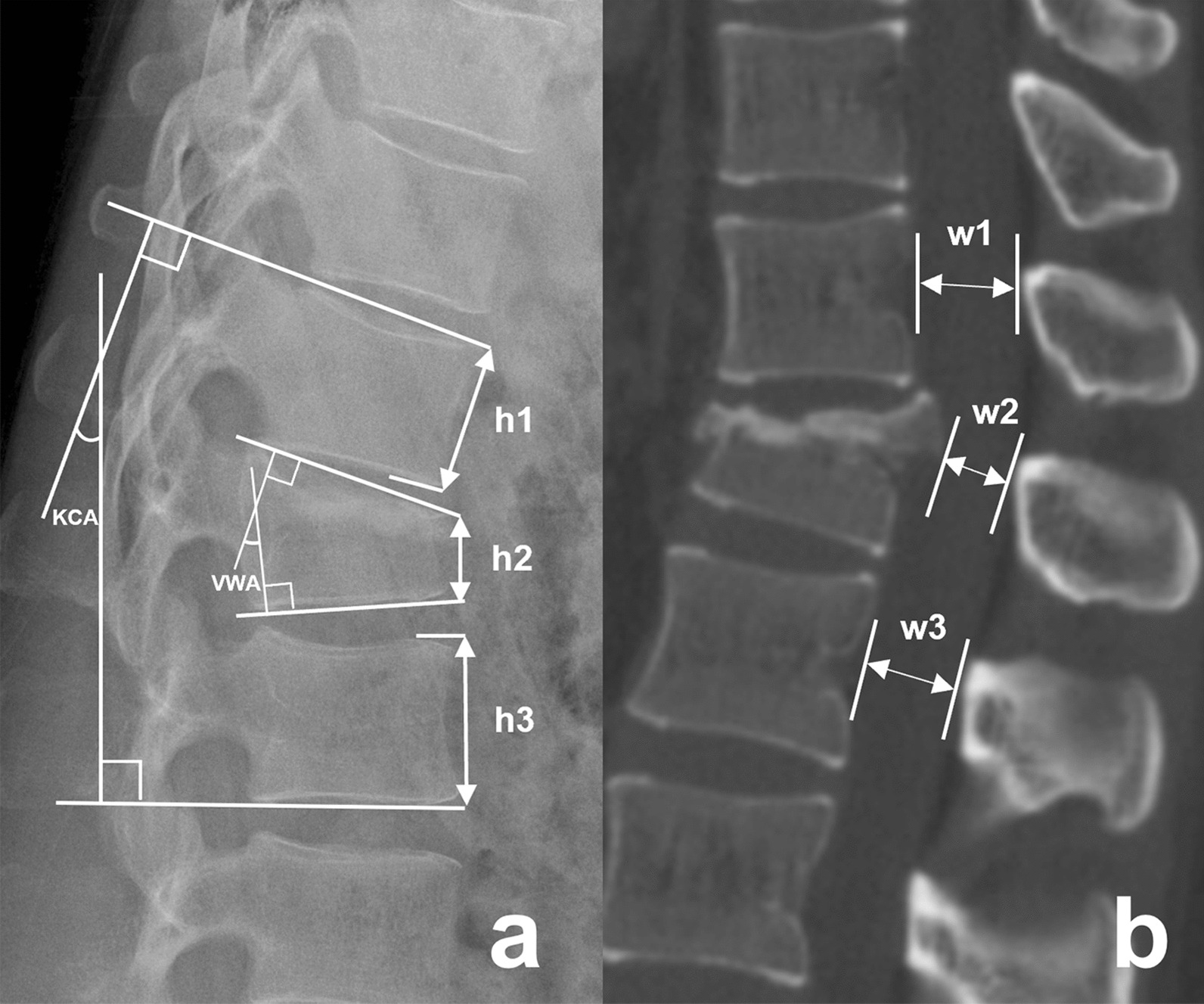
Measurement of radiologic evaluation indicators.** a** AVHR = *h*2/ [(*h*1 + *h*3)/2] × 100%, the KCA was measured between the upper endplate of the adjacent superior vertebra of the injured vertebra and the lower endplate of the adjacent inferior vertebra of the injured vertebra, the VWA was measured between the upper endplate of the injured vertebra and the lower endplate of the injured vertebra. **b** SCER = *w*2/ [(*w*1 + *w*3)/2] × 100%

Radiologic evaluation indicators included the anterior vertebral height ratio (AVHR), kyphosis Cobb angle (KCA), vertebral wedge angle (VWA) and spinal canal encroachment rate (SCER). The AVHR, KCA and VWA were measured on lateral images of the thoracolumbar spine (Fig. 2a). The SCER was measured on CT images (Fig. 2b). Anteroposterior and lateral radiographs, as well as CT, were obtained for the thoracolumbar spine before surgery, at 1 week and 3 months after surgery, and during the final follow-up at our hospital for all patients. Additionally, MRI of the thoracolumbar spine were conducted before surgery and at 1 week after surgery for all patients. The effect of correction was compared and analysed in terms of AVHR, KCA, VWA and SCER. Changes in the corrective effect were evaluated on all lateral radiographs and CT of the thoracolumbar spine after the surgery.

Perioperative indicators included the time from admission to surgery, operation time, intraoperative bleeding volume, time to ambulation after surgery and length of hospital stay.

Clinical assessment indicators included the visual analogue scale (VAS) score, Oswestry Disability Index (ODI) score and complications (spinal cord or nerve root injury, wound haematoma, infection, internal fixation failure, etc.). Pain from the lower back was evaluated using the VAS score and functional outcome was evaluated using the ODI score. The VAS score and ODI score were calculated preoperatively, 1 week and 3 months postoperatively and at the last follow-up. Complications, including intraoperative and postoperative complications, were recorded. Loosening, breakage and pulling out of internal fixation (including screw, rod and locking cap) were defined as failure.

All measured and evaluated data were performed by two experienced physicians in spine surgery respectively. The final values were determined as the average of the measurements performed by the two physicians.

### Statistical analyses

All of the statistical analyses were conducted with SPSS 26.0 (IBM, New York, USA). The categorical variables that were reported as numbers were compared by using the *χ*^2^ test or Fisher’s exact test. The variables with continuous data were reported as the mean ± standard deviation. Comparisons between two groups were evaluated by independent sample t tests, and comparison of before–after changes in each group was using paired-samples t test. A *P* value < 0.05 indicated statistical significance.

## Results

### Demographic data

In the study, a total of 100 patients were recruited with an average age of 46.11 ± 12.3 years. Fifty-one patients underwent fixation with 6 MSs (MS group) and 49 patients underwent fixation with hybrid 4 fixed axial screws and 2 polyaxial screws (HS group). In terms of sex, age, cause of injury, fracture level, AO classification, TLICS score, LSC score, number of spinal canal encroachment and time of last follow-up were not statistically different between the MS group and the HS group (*P* > 0.05, Table 1).

**Table 1:** Comparisons of demographic data between the two groups

	MS group (*N* = 51)	HS group (*N* = 49)	*p* Value
*Sex*			0.387
Male	29 (56.9%)	32 (65.3%)	
Female	22 (43.1%)	17 (34.7%)	
Age (year)	44.9 ± 12.6	47.3 ± 12.1	0.336
*Cause of injury*			0.489
Falling from height	19 (37.3%)	18 (36.7%)	
Fall	25 (49.0%)	20 (40.8%)	
Traffic accidents	7 (13.7%)	11 (22.4%)	
*Fracture level*			0.549
*T*11	1 (2.0%)	0 (0.0%)	
*T*12	16 (31.4%)	11 (22.4%)	
*L*1	17 (33.3%)	21 (42.9%)	
*L*2	17 (33.3%)	17 (34.7%)	
*AO classification*			0.425
*A*3	39 (76.5%)	34 (69.4%)	
*A*4	12 (23.5%)	15 (30.6%)	
*TLICS score*			0.410
4	14 (27.5%)	10 (20.4%)	
5	37 (72.5%)	39 (79.6%)	
*LSC score*			0.694
3	3 (5.9%)	4 (8.2%)	
4	6 (11.8%)	8 (16.3%)	
5	14 (27.5%)	17 (34.7%)	
6	20 (39.2%)	13 (26.5%)	
7	8 (15.7%)	7 (14.3%)	
Number of spinal canal encroachment	24 (47.1%)	19 (38.8%)	0.403
Time of last follow-up (month)	13.3 ± 1.2	13.1 ± 1.4	0.420

MS group, 6 monoplanar screws fixation; HS group, hybrid 4 fixed axial screws and 2 polyaxial screws fixation

### Perioperative indicators

No statistically significant differences in the time from admission to surgery, operation time, intraoperative bleeding volume, time to ambulation after surgery or length of hospital stay were found between the MS and HS groups (*P* > 0.05, Table 2).

**Table 2:** Comparisons of perioperative indicators between the two groups

	MS group	HS group	*p* Value
Time from admission to surgery (day)	3.1 ± 1.3	3.2 ± 1.2	0.735
Operation time (min)	81.8 ± 10.1	79.5 ± 8.7	0.217
Intraoperative bleeding volume (ml)	66.3 ± 14.7	62.0 ± 11.0	0.107
Time of ambulation after surgery (day)	3.3 ± 0.8	3.2 ± 0.7	0.328
Length of hospital stay (day)	9.6 ± 1.8	9.8 ± 1.6	0.585

### Radiologic and clinical outcomes

As shown in Table 3, no significant differences in the AVHR, KCA, VWA or SCER were found between the two groups preoperatively (*P* > 0.05). One week postoperatively, 3 months postoperatively and at the last follow-up, the AVHR, KCA, VWA and SCER were obviously improved in the two groups. A significant difference between the preoperative and postoperative values were found at every time point (*P < 0.05). The MS group had better correction in the AVHR, KCA and VWA than the HS group after surgery (*P < 0.05). During the follow-up period, correction loss of AVHR, KCA and VWA was found between two groups and correction loss was clearly observed in the HS group at the last follow-up (*P < 0.05). The MS group presented greater improvement in the SCER than the HS group at the last follow-up (*P < 0.05).

**Table 3:** Comparisons of radiologic evaluation indicators between the two groups

	MS group	HS group	*p* Value
*AVHR (%)*			
Preoperation	69.4 ± 7.5	69.8 ± 8.8	0.819
1 week postoperatively	96.9 ± 2.6	95.0 ± 3.1	0.001*
3 months postoperatively	96.2 ± 2.6	94.2 ± 3.2	0.001*
Last follow-up	94.8 ± 2.4	92.4 ± 3.3	0.000*
Correction loss (3 months postoperatively)	0.6 ± 0.5	0.8 ± 0.5	0.124
Correction loss (last follow-up)	2.1 ± 1.0	2.6 ± 1.0	0.037*
*KCA (◦)*			
Preoperation	19.9 ± 2.1	19.7 ± 2.1	0.626
1 week postoperatively	4.8 ± 2.2	5.7 ± 1.5	0.013*
3 months postoperatively	5.2 ± 2.3	6.3 ± 1.4	0.004*
Last follow-up	6.2 ± 2.2	7.6 ± 1.8	0.001*
Correction loss (3 months postoperatively)	0.4 ± 0.6	0.6 ± 0.7	0.181
Correction loss (last follow-up)	1.5 ± 1.0	1.9 ± 1.2	0.038*
*VWA (◦)*			
Preoperation	17.8 ± 2.5	18.1 ± 2.5	0.630
1 week postoperatively	3.9 ± 1.4	4.7 ± 1.6	0.010*
3 months postoperatively	4.2 ± 1.5	5.1 ± 1.6	0.003*
Last follow-up	4.9 ± 1.7	6.1 ± 1.7	0.001*
Correction loss (3 months postoperatively)	0.3 ± 0.4	0.4 ± 0.6	0.154
Correction loss (last follow-up)	1.0 ± 0.8	1.4 ± 0.8	0.025*
*SCER (%)*			
Preoperation	26.1 ± 9.4	25.8 ± 7.3	0.908
1 week postoperatively	19.5 ± 8.5	19.8 ± 7.0	0.892
3 months postoperatively	19.2 ± 8.4	19.7 ± 6.9	0.837
Last follow-up	18.4 ± 8.5	19.5 ± 7.0	0.644
Encroachment improvement (3 months postoperatively)	0.3 ± 0.4	0.2 ± 0.2	0.141
Encroachment improvement (last follow-up)	1.1 ± 1.1	0.4 ± 0.3	0.004*

AVHR, anterior vertebral height ratio; KCA, kyphosis Cobb angle; VWA, vertebral wedge angle; SCER, spinal canal encroachment rate

*Statistically significant difference (*p* < 0.05) between the two groups at that time point

**Fig. 3:**
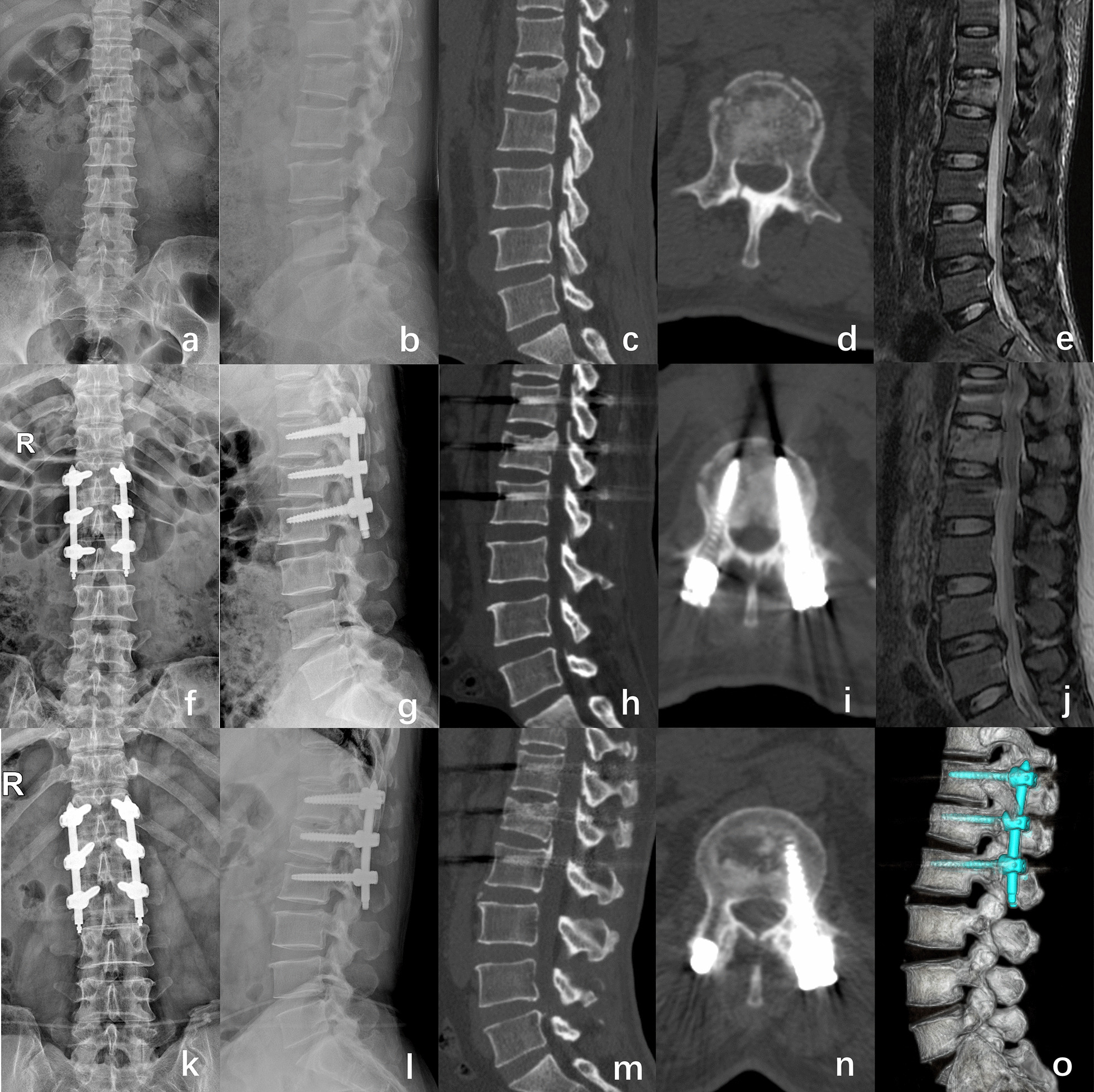
A 46-year-old female patient with L1 burst fracture was treated with MSs fixation. **a–d** Preoperative lumbar radiographs (**a**, **b**) and CT (**c**, **d**) demonstrated a L1 AO type A3 fracture with spinal canal encroachment. **e** MRI showed a fresh fracture of the L1 vertebrae. **f–i** One-week postoperative radiographs (**f**–**g**) and CT (**h**–**i**) showed satisfactory injury vertebral height restoration and kyphosis correction. **j** One-week postoperative MRI show the spinal canal encroachment decreased. **k–l** Radiography at the last follow-up at 12 months after surgery showed that the internal fixation systems were stable with good positioning and good morphology. **m–o** And CT showed that spinal canal encroachment was remarkably relieve

Compared to those preoperatively, the postoperative VAS and ODI scores were significantly improved in all the patients in both groups (*P < 0.05), and the scores collected at each follow-up visit did not differ significantly between the two groups (*P* > 0.05) (Table 4). In the HS group, two cases of internal fixation failure were observed at the last follow-up and the overall failure rate was 4.1%. There was 1 case of rod loosening that developed 13 months after surgery, and one case of screw breakage developed 12 months postoperatively. In two cases, good fracture healing and correction maintenance were found. After implant removal no more problems occurred. There were no severe complications such as spinal cord or nerve root injury, wound haematoma or infection, in either group. Typical case images of the MSs fixation were shown in (Fig. 3).

**Table 4:** Comparisons of clinical assessment indicators between the two groups

	MS group	HS group	*p* Value
*VAS score*			
Preoperation	7.8 ± 0.7	7.9 ± 0.8	0.926
1 week postoperatively	2.6 ± 0.7	2.6 ± 0.7	0.864
3 months postoperatively	1.1 ± 0.7	1.2 ± 1.0	0.456
Last follow-up	0.6 ± 0.7	0.7 ± 0.7	0.518
*ODI score (%)*			
Preoperation	85.3 ± 5.3	86.3 ± 4.4	0.274
1 week postoperatively	59.6 ± 3.9	60.7 ± 3.1	0.115
3 months postoperatively	11.7 ± 1.8	12.2 ± 2.4	0.223
Last follow-up	5.3 ± 2.0	5.4 ± 2.1	0.748

VAS, visual analogue scale; ODI, Oswestry Disability Index

## Discussion

The objective of surgical treatment of TTBFs is effective fixation with pedicle screws to restore the injured vertebral height, correct kyphosis, and prevent late-onset kyphosis of the spine and secondary nerve compression [28]. In a clinical setting, PSISF that can be used to provide effective support of the anterior, middle and posterior columns for the vertebral bodies has become one of the most commonly used surgical options for treating TTBFs in patients without neurological function deficits [29–31].

As PSISF requires 6-screw fixation, the insertion of longitudinal rods would become difficult if the 3 ipsilateral screws were not highly aligned, especially using fixed axial screws [22]. Thus, proper choice of pedicle screw appears to be critical for the treatment of TTBFs. There are several options of pedicle screws for PSISF, including fixed axial screw, polyaxial screw, and MS [32]. Polyaxial screw has been used widely in PSISF [33]. Benefiting from the well-orchestrated coupling device, polyaxial screw can increase the capacity of angular motion and offer convenience for rod implantation without increasing overmuch stress [17, 34]. However, polyaxial screw reduce the compression and bending strength in the sagittal plane, which results in insufficient restoration of vertebral height, inadequate correction of kyphotic and inadequate maintenance of rigid fixation [17, 18].

Percutaneous MSs and HSs fixation were proposed to solve the abovementioned problems of polyaxial screw [23, 35]. Because MS is fixed in the sagittal view and allows free swivelling in the coronal view, 6 MSs fixation do not compromise the rigidity in the sagittal view and allow smooth installation of the connecting rod [35]. Owing to fixed axial screw endows stronger leverage, HSs fixation can better improve the correction, stability of the instrumentations and buckling and compressive strengths than polyaxial screw fixation alone. The injured vertebra fixation with 2 polyaxial screws convenient for rod implantation [33, 34]. Whether there was a difference between the two in terms of specific clinical outcome was not reported. To compare the clinical outcomes of MSs versus HSs fixation for TTBFs, we conceived and designed this study.

In this study we observed that the AVHR, KCA, VWA and SCER were significantly improved in both groups immediately after surgery, which suggested that both MSs and HSs fixation are safe and effective approaches in the treatment of TTBFs. This is consistent with previous studies [23, 35, 36]. We also found that the MS group had better correction in terms of the AVHR, KCA and VWA after surgery. Although there were no significant differences in the short‐term postoperative outcomes, we found that the MS group had less correction loss in terms of the AVHR, KCA and VWA and greater improvement in the SCER than the HS group at the last follow-up. We contemplated that the reasons were related to the following factors. In the MS group, one is that MS had strong strength in the sagittal plane to achieve effective reduction, immobilization, and stabilization, which thereby ensured long-term stability and potentially decreased the spinal canal encroachment. The other is that intermediate fixation with MS that were inserted parallel to the upper endplate of the injured vertebra can circumvent the anterior vertebral fracture regions and use relatively longer screws, allowing better restoration of the anterior vertebral height and the upper endplate of the injured vertebra. Similarly, a clinical study by Huang et al. [37] indicated that MSs fixation achieved better correction effect and less correction loss than polyaxial screw fixation alone. According to Ye et al. [38] MS probably a superior option to reduce the incidence of degeneration of the injured vertebral adjacent segment than fixed axial screw.

Additionally, no significant differences in any of the perioperative indicators were found between the two groups. Our results showed that both MSs and HSs fixation are work well, highly efficient and less traumatic. Postoperative VAS and ODI scores of all patients had improved obviously, indicating a significant decrease in pain. The pain scores were further decrease between the two groups during an extended follow-up; the VAS and ODI scores were no significant difference in both group at the last follow-up. This suggests that the two surgical methods showed favourable clinical results. Furthermore, two cases of instrumentation failure were observed at the last follow-up in the HS group and not in the MS group. This indicates that the MSs fixation system could sustain higher loads than the HSs fixation system and have advantages in the prevention of internal fixation failure. Yin et al. [39] observed that HSs fixation can cause more correction loss but no instrumentation failure events. According to the study by Liu et al. [19], fixed axial screw was implanted at the lowest segment led to a greater predisposition to adjacent segmental degeneration, particularly at the one level above the injured vertebra.

To the best of our knowledge, this is the first study to compare MSs versus HSs fixation for the treatment of TTBFs. Our study provides evidence that both MSs and HSs fixation can be used for treating TTBFs. MSs fixation can achieve better correct results, less correction loss, greater improvement of the SCER and fewer instrumentation failures.

### Limitations of this study

There were several limitations of this study. First, its retrospective design and lack of randomization could potentially lead to selection bias. Therefore, our findings should be confirmed in additional prospective studies. Second, the study sample size was relatively small. Thus, multicentre relevant studies are needed. Third, we did not assess results of correction loss, functional outcome, and degeneration of adjacent segment of the injured vertebra after removing the internal fixation. As such, further research in this area is warranted.

## Conclusions

Both MSs and HSs fixation are effective treatments for TTBFs and have comparable clinical outcomes. In contrast, MSs fixation can improve the correction effect, better improve the SCER, and further reduce correction loss as well as reduce the incidence of instrumentation failure. Therefore, MSs fixation might be a better option for treating TTBFs in patients without neurological deficits.

## Data Availability

The datasets used and analysed during the current study are available from the corresponding author on reasonable request.

## Abbreviations

MS: Monoplanar pedicle screw
HSs: Hybrid fixed axial and polyaxial pedicle screws
PSISF: Percutaneous short-segment intermediate screw fixation
TTBFs: Traumatic thoracolumbar burst fractures
AVHR: Anterior vertebral height ratio
KCA: Kyphosis Cobb angle
VWA: Vertebral wedge angle
SCER: Spinal canal encroachment rate
VAS: Visual analogue scale
ODI: Oswestry Disability Index
PPSF: Percutaneous pedicle screw fixation
AO classification: AO spine injury classification
TLICS: Thoracolumbar injury classification and severity
LSC: Load sharing classification
ASIA: American Spinal Injury Association
